# Mechanisms underlying the anti-aging activity of bergamot (*Citrus bergamia*) extract in human red blood cells

**DOI:** 10.3389/fphys.2023.1225552

**Published:** 2023-06-30

**Authors:** Alessia Remigante, Sara Spinelli, Elisabetta Straface, Lucrezia Gambardella, Marina Russo, Giovanna Cafeo, Daniele Caruso, Giuseppe Falliti, Paola Dugo, Silvia Dossena, Angela Marino, Rossana Morabito

**Affiliations:** ^1^ Department of Chemical and Biological, Pharmaceutical and Environmental Sciences, University of Messina, Messina, Italy; ^2^ Biomarkers Unit, Center for Gender-Specific Medicine, Istituto Superiore di Sanità, Rome, Italy; ^3^ Complex Operational Unit of Clinical Pathology of Papardo Hospital, Messina, Italy; ^4^ Institute of Pharmacology and Toxicology, Paracelsus Medical University, Salzburg, Austria

**Keywords:** flavonoid fraction, bergamot (citrus bergamia), peel and/or juice extract, oxidative stress, aging, red blood cells, band 3 anion exchanger

## Abstract

**Introduction:** Aging is a process characterised by a decline in physiological functions. Reactive species play a crucial role in the aging rate. Due to the close relationship between aging and oxidative stress, functional foods rich in phytochemicals are excellent candidates to neutralise age-related changes.

**Aim:** This investigation aims to verify the potential protective role of bergamot (*Citrus bergamia*, *Femminello* cultivar) peel and juice extract in a model of aging represented by human red blood cells (RBCs) exposed to D-Galactose (DGal).

**Methods:** Bergamot peel and juice extracts were subjected to RP-HPLC/PDA/MS for determination of their composition in bioactive compounds. Markers of oxidative stress, including ROS production, thiobarbituric acid reactive substances (TBARS) levels -a marker of lipid peroxidation, oxidation of total protein sulfhydryl groups, as well as the expression and anion exchange capability of band 3 and glycated haemoglobin (A1c) production have been investigated in RBCs treated with D-Gal for 24 h, with or without pre-incubation for 15 min with 5 μg/mL peel or juice extract. In addition, the activity of the endogenous antioxidant system, including catalase (CAT) and superoxide dismutase (SOD), as well as the diversion of the RBC metabolism from glycolysis towards the pentose phosphate pathway shunt, as denoted by activation of glucose-6-phosphate dehydrogenase (G6PDH), have been explored.

**Results:** Data shown here suggest that bergamot peel and juice extract i) prevented the D-Gal-induced ROS production, and consequently, oxidative stress injury to biological macromolecules including membrane lipids and proteins; ii) significantly restored D-Gal-induced alterations in the distribution and ion transport kinetics of band 3; iii) blunted A1c production; iv) effectively impeded the over-activation of the endogenous antioxidant enzymes CAT and SOD; and v) significantly prevented the activation of G6PDH.

**Discussion:** These results further contribute to shed light on aging mechanisms in human RBCs and identify bergamot as a functional food rich in natural antioxidants useful for prevention and treatment of oxidative stress-related changes, which may lead to pathological states during aging.

## 1 Introduction

Aging is a biological process that results in a progressive and non-reversible decline in the physiological functions of all body organs and is caused by damage accumulation with concomitant elevation of oxidative stress (Fukagawa, 1999; Akki et al, 2018; Akki et al, 2019; Ferrera et al, 2021). In 1972, Denham Harman (Harman, 1972) postulated the free radical theory of aging, which points to accumulation of reactive species as the underlying reason for the oxidation of the biological macromolecules and consequent cell injury, thereby explaining the alterations in cell functions during natural aging (Junqueira et al, 2004; Remigante and Morabito, 2022).

Aerobic cell metabolism requires oxygen as the final electron acceptor of oxidation reactions and reactive oxygen species (ROS) represent a byproduct of this process. The physiological role of red blood cells (RBCs) is the transport of respiratory gases from the lungs to the tissues and *vice versa*, in order to supply the cells with oxygen. In the blood stream, RBCs are continuously exposed to both endogenous and exogenous reactive species able to injury their structure, thus impairing their physiology and functionality (Rizvi and Maurya, 2007; Remigante et al, 2021a; Fujii et al, 2021; Arrigo et al, 2023). Un-neutralized reactive species react with both RBC plasma membrane lipids and proteins and promote oxidative alterations at level of lipids (lipid peroxidation) and proteins (protein oxidation) and/or fragmentation, respectively (Avitabile et al, 2020). Alternatively, the accumulation of reactive species results in the induction of glycation reactions, which leads to the increased endogenous production of advanced glycation end products (AGEs) (Moldogazieva et al, 2019). Non-enzymatic glycation of membrane glycoproteins and/or haemoglobin as well as their gradual accumulation within RBCs can account for altered rheologic properties of RBCs (Turk et al, 1998). In RBCs, oxidative stress-related aging is accompanied by a decrease in cell volume and hemoglobin content and an increase in cell density (Pandey and Rizvi, 2010). These alterations are correlated with a loss of cholesterol and phospholipids, resulting in a decrease in the surface area that reflects a loss of membrane lipids and protein constituents. The reduction of surface area could be explained by a gradual membrane blebbing and vesiculation (Willekens et al., 2008), both accelerated in aged RBCs (Pandey and Rizvi, 2011). In addition, these changes might limit the ability of the RBCs to maintain the highly deformable biconcave shape necessary to pass through the narrow capillaries, thus contributing to their removal from circulation (Shiga et al, 1990; Pretorius, 2018).

One of the major goals of RBC redox regulation results in protection of their main plasma membrane protein, band 3 (SLC4A1/AE1) (Abbas et al, 2018). Band 3 is an integral trans-membrane protein that plays different functions: 1) the maintenance of anion homeostasis (Reithmeier et al, 2016); 2) the binding between plasma membrane lipids and cytoskeletal proteins, reflecting on cell shape (De Franceschi et al, 2012; Vallese et al, 2022; Spinelli et al, 2023); 3) the docking, at level of the N-terminal cytosolic domain that protrudes into the cytosol, of a series of structural proteins and glycolytic enzymes (e.g., glyceraldehyde 3-phosphate dehydrogenases: GAPDH) (Campanella et al, 2008; Puchulu-Campanella et al, 2013), as well as cytosolic proteins such as haemoglobin (Zhang et al, 2003). Moreover, haemoglobin, in addition to its central role of carrying oxygen from the lungs to peripheral tissues, may serve as oxygen sensor, in order to appropriately link band 3 to regulation of the RBC metabolism (Ellsworth, 2000; Puchulu-Campanella et al, 2013). In fact, several studies have provided compelling, albeit indirect, evidence that, when not encumbered by deoxy-hemoglobin, the N-terminal of band 3 can bind to and, in turn, inhibit the glycolytic enzyme GAPDH (Issaian et al, 2021). Oxygen-dependent metabolic modulation is mediated by the competitive binding of deoxy-hemoglobin and glycolytic enzymes to the band 3 cytosolic domain. When oxidant stress is high, GAPDH enzyme is bound to band 3 and thereby inhibited (Reisz et al, 2016). In this context, RBCs favor glucose oxidation through the pentose phosphate pathway (PPP), in order to generate the reducing cofactor NADPH and fuel endogenous antioxidant systems. On the other hand, when oxidant stress is low, haemoglobin is deoxygenated and binds to the band 3 N-terminus, which in turn favors the release of glycolytic enzymes from the membrane to promote the generation of energy in the form of ATP and NADH through glycolysis (Castagnola et al, 2010). This balance may be dysregulated by early RBCs aging and/or increased reactive oxygen species (ROS) levels, thus depriving RBCs of their crucial metabolic plasticity and leading to their removal from the blood circulation (Kuhn et al, 2017). Therefore, the regulation of the balance between glycolysis and PPP is essential and enables RBCs to counteract oxidative insults impacting on their cell structures/functions. In addition, unusual levels of reactive species could be the common denominator in the development and progression of different aging-related acute and/or chronic pathologies, although the precise mechanisms contributing to oxidative stress-induced injury are still poorly clarified (Radi et al, 2014; Bo-Htay et al, 2018; Martinelli et al, 2020; Emanuelli et al, 2022; Guo et al, 2022; Remigante and Morabito, 2022).

To contain oxidative stress effects, RBCs possess an excellent cytosolic antioxidant machinery composed of non-enzymatic as well as enzymatic antioxidants, which represent an effective antioxidant equipment to protect RBCs themselves along with other body cells and tissues (Zabinski et al, 2000; Inal et al, 2001). The use of natural secondary metabolites such as polyphenol-rich extracts with antioxidant properties could be an excellent and workable alternative for supporting the integrated antioxidant system (Jacob, 1995; Lunceford and Gugliucci, 2005; Dorta et al, 2008; Kouka et al, 2017; Xu et al, 2019; Remigante et al, 2022a; Remigante et al, 2022b). In this regards, special attention has been paid to the potential health benefits of the flavonoid fraction of the bergamot (*Citrus bergamia*) (Russo et al, 2016).

Bergamot is a small tree belonging to Rutaceae family that is mainly cultivated in a specific area of the region of Calabria (Italy) known to ensure a microclimate suitable for its growth. The essential oil of bergamot, which is obtained from the fruit peel, has been extensively characterized and used in cosmetic and food industries, whereas the bergamot by-products, such as the pulp and juice, have been only recently evaluated for their beneficial properties, which include cholesterol reduction, antioxidant and anti-inflammatory effects (Ferlazzo et al, 2015; Russo et al, 2015; Carresi et al, 2016; Lauro et al, 2016; Musolino et al, 2019). This reassessment may lead to reduction in the disposal costs of industrial processes and gain of a good source of nutraceuticals, thus representing an economic advantage (Russo et al, 2021). To date, no scientific study evaluating the anti-aging properties of peel and/or juice extracts from bergamot in human RBCs has been reported.

The long-term exposure to high doses of D-Galactose (D-Gal) represents a good experimental model of natural aging (Azman and Zakaria, 2019). Thus, the present investigation aims to identify the potential beneficial effects of bergamot peel and juice extracts from *Femminello* cultivar on the molecular mechanisms underlying natural aging in RBCs, including oxidative damage, glycation events and activation of the endogenous enzymatic defense system. To this aim, juice and peel extracts from bergamot fruits belonging to *Femminello* cultivar were subjected to RP-HPLC/PDA/MS for determination of their precise composition in bioactive compounds and their effects were evaluated in a D-Gal-induced model of aging in human RBCs.

## 2 Materials and methods

### 2.1 Materials and samples for analytical determination of bioactive molecules

Water, formic acid, ethanol, acetonitrile and methanol were obtained from Merck Life Science (Merck KGaA, Darmstadt, Germany). The standard compounds ferulic acid, synapic acid, eriocitrin, narirutin, and neohesperidin were obtained from Extrasynthese (Genay Cedex, France). Apigenin 6,8-di-*C*-glucoside, diosmetin 6,8-di-*C*-glucoside, naringin, brutieridin, limonin glucoside, nomilin glucoside, nomilinic acid glucoside, limonin, melitidin, nomilin and neoeriocitrin were previously isolated in our laboratory. Bergamot (*Citrus bergamia*) peels used in this research belong to *Femminello*cultivar and the experimental protocol has been performed with peels obtained from two fruits. Fruits were collected at the same stage of ripeness from trees grown in Calabria (Melito di Porto Salvo, Reggio Calabria, Italy) on January 2023. Fruits were washed, dried and stored at +4°C, then peeled and squeezed. Peels were dried at 25°C for 48 h.

The juice was subjected to RP-HPLC analysis without any pre-treatment. On the other hand, a solvent extraction procedure was performed on peel samples before RP-HPLC analysis. The bioactive compounds extraction procedure was previously validated by our group (Russo et al, 2015). Briefly, 10 g of samples were extracted with 100 mL of methanol for three times. The extract was subjected to chromatographic analysis. The juice and the peel extract were analysed in triplicate.

### 2.2 Determination of bioactive compounds using RP-HPLC/PDA/MS

A Shimadzu Prominence LC-20A system (Shimadzu, Milan, Italy) was employed to carry out HPLC analyses. The HPLC instrument was equipped with SPD-M20A UV and HPLCMS-2020 detectors. The analytical procedure was previously validated by our group (Russo et al, 2014). This analytical method allowed to quantify the bioactive molecule content in juice and bergamot peel samples. Figure of merits were calculated in accordance with the EURACHEM guidelines (Group, 1998).

### 2.3 Solutions and chemicals used to RBC sample processing

Chemicals used to perform experiments were purchased from Sigma (Milan, Italy). With regard to stock solutions, 4, 4′-diisothiocyanatostilbene-2, 2′-disulfonate (DIDS, 10 mM) was prepared in dimethyl sulfoxide (DMSO); D-Galactose (D-Gal, 1 M) was prepared in distilled water and N-ethylmaleimide (NEM, 310 mM) was prepared in ethanol. H_2_O_2_ experimental solution was obtained by dilution in distilled water from a 30% w/w stock solution. Ethanol never exceeded 0.001% v/v in the experimental solutions and was previously tested on RBCs to exclude haemolysis. Peel extract obtained as described above and juice devoid of the fibrous part were poured into freeze-drying flasks and placed into the vacuum chamber, frozen at −50°C, and then freeze-dried for 72 h (BenchTop K Series Freeze Dryers, VirTis Gardiner, United States). The concentrated peel extract required a dilution 1:100 w/v with distilled water prior to use in experiments.

### 2.4 Preparation of RBCs

Blood needed for the planned experiments was withdrawn from healthy volunteers (age 25–45 years) upon their informed consent. Whole blood samples, put in test tubes containing ethylenediaminetetraacetic acid (EDTA) as anticoagulant, were repeatedly washed in isotonic solution (NaCl 150 mM, 4-(2-hydroxyethyl)-1-piperazineethanesulfonic acid (HEPES) 5 mM, glucose 5 mM, pH 7.4, osmotic pressure 300 mOsm/kgH_2_O; centrifugation with Neya 16R, 1,200 × g, 5 min) to eliminate plasma along with buffy coat. As a further step, thus obtained RBCs were put in isotonic solution at the haematocrit index requested by each protocol.

### 2.5 Haemolysis measurement

To determine the % haemolysis, RBCs (35% haematocrit) were treated with or without peel or juice extract in isotonic solution for 15 min at 37°C and then processed according to Remigante and collaborators (Remigante et al, 2022b). Haemoglobin absorbance was determined at 405 nm wavelength after subtracting the absorbance of blank (0.9% v/v NaCl solution).

### 2.6 Determination of intracellular reactive oxygen species (ROS)

The ROS levels were evaluated by the cell-permeable indicator 2′, 7′-dichlorofluorescein diacetate (H2DCFDA, D6883, Sigma-Aldrich), according to the manufacturer’s instructions, with slight modifications. Red blood cells were exposed to 100 mM D-Gal for 24 h at 25°C with or without pre-incubation with different concentrations of peel or juice extract. As the positive control, RBCs were incubated with H_2_O_2_. ROS formation was determined by a fluorescence microplate reader (Onda Spectrophotometer, UV-21) at excitation and emission wavelengths of 485 nm and 535 nm, respectively, after subtracting the background fluorescence. Results are expressed in %.

### 2.7 Measurement of thiobarbituric-acid-reactive substances (TBARS) levels

TBARS levels were detected as described by Mendanha and co-authors (Mendanha et al, 2012), with minor modifications. Red blood cells were suspended at 20% haematocrit and incubated with or without different concentrations of peel or juice extract. Next, samples were incubated in 100 mM D-Gal-containing solution and then addressed to quantification of TBARS levels obtained by subtracting 20% of the absorbance at 453 nm from that one at 532 nm (Onda Spectrophotometer, UV-21). Finally, the results were reported as µM TBARS levels.

### 2.8 Measurement of total sulfhydryl group (-SH) content

Measurement of total -SH groups was performed according to Aksenov and Markesbery technique (Aksenov and Markesbery, 2001) with minor modifications. Red blood cells, suspended at 35% haematocrit, were incubated with or without peel or juice extract at different concentrations and successively exposed to D-Gal. To obtain a complete oxidation of total -SH groups, the treatment with NEM was used as the positive control. After that, samples were spectrophotometrically read at 412 nm (Onda spectrophotometer, UV-21). Data were normalised to protein content and results reported as μM TNB/mg protein.

### 2.9 Analytical cytology

Band 3 expression levels were detected according to Straface and collaborators (Straface et al, 2011). The analysis was performed by an Olympus BX51 Microphot fluorescence microscope or by a FACScan flow cytometer (Becton Dickinson, Mountain View, CA, United States) equipped with a 488 nm argon laser on left untreated RBCs or after their exposure to D-Gal, with or without pre-incubation with peel or juice extract. The median values of fluorescence intensity histograms were used to provide a semi-quantitative analysis.

### 2.10 SO_4_^2−^ uptake measurement

#### 2.10.1 Control condition

To establish the anion exchange via band 3, SO_4_^2−^ uptake was measured as formerly described (Romano and Passow, 1984; Galtieri et al, 2002; Romano et al, 2002; Morabito et al, 2013; Morabito et al, 2016; Morabito et al, 2018; Morabito et al, 2019a; Morabito et al, 2020a; Remigante et al, 2020; Perrone et al, 2023). Shortly, this procedure allows for the determination of the kinetic of transport and amount of SO_4_^2−^ internalized by RBCs by turbidimetric analysis after precipitation of the cell content with BaCl_2_. In particular, each sample was addressed to the spectrophotometer (UV-21, Onda Spectrophotometer, Carpi, Modena, Italy, 425 nm wavelength) and the obtained absorbance was successively converted to (SO_4_^2−^) L cells × 10^–2^ based on a calibrated standard curve previously established by precipitating known SO_4_^2−^ concentrations. Sulphate concentration was needed to quantify the rate constant of SO_4_^2−^ uptake (min^−1^). To this end the following equation was used: Ct = C∞ (1 − e−rt) + C0 (Ct, C∞, and C0 indicate the intracellular SO_4_^2−^ concentrations measured at time t, ∞, and 0, respectively, e indicate the Neper number (2.7182818), r indicates the rate constant accounting for the process velocity, t is the time at which the SO_4_^2−^ concentration was measured). The rate constant is useful to monitor the anion exchange process, as it specifically represents the inverse of the time needed to reach ∼63% of total SO_4_^2−^ intracellular concentration. The parameter (SO_4_^2−^) L cells × 10^–2^ reported in figures corresponds to the micromolar concentration of SO_4_^2−^ internalized by 5 mL RBCs at 3% haematocrit.

#### 2.10.2 Experimental conditions

After 15 min pre-incubation with or without peel or juice extract, RBCs (3% haematocrit) were incubated with D-Gal and then centrifuged (Neya 16R, 4°C, 1,200 × g, 5 min) to discard the supernatant and to suspend RBCs in SO_4_^2−^ medium. Similarly to what described for control conditions, the rate constant for SO_4_^2−^ uptake was quantified.

### 2.11 Advanced glycation end products (AGEs): measurement of glycated haemoglobin (A1c) levels

The glycated haemoglobin content (%A1c) was measured as described by Sompong and collaborators (Sompong et al, 2015) with minor modifications. To this end, RBCs pre-exposed or not to peel or juice extract, were incubated with D-Gal and successively addressed to spectrophotometrically analysis (BioPhotometer Plus, Eppendorf, Manchester, United Kingdom, 610 nm wavelength). Finally, A1c levels (%) were determined from a standard curve obtained from known A1c doses.

### 2.12 Assessment of the endogenous antioxidant activity

#### 2.12.1 Superoxide dismutase (SOD) activity assay

Superoxide dismutase (SOD) activity was evaluated by a specific assay kit (CS0009, Sigma-Aldrich, Milan, Italy), according to the manufacturer’s instructions, with slight modifications. Red blood cells were treated with D-Gal, with or without pre-incubation with peel or juice extract. As the positive control, cells were incubated with H_2_O_2_. Superoxide dismutase activity was determined by reading the absorbance at 450 nm wavelength (Fluostar Omega, BMG Labtech, Ortenberg, Germany) after subtracting the background.

#### 2.12.2 Catalase (CAT) activity assay

Catalase activity was evaluated by the catalase assay kit (MAK381, Sigma-Aldrich, Milan, Italy), according to the manufacturer’s instructions, with slight modifications. Red blood cells were treated with D-Gal with or without pre-incubation with peel or juice extract. As the positive control, cells were treated with H_2_O_2_. Catalase activity was determined by reading the absorbance at 570 nm wavelength (Fluostar Omega, BMG Labtech, Ortenberg, Germany) after subtracting the background.

#### 2.12.3 Glucose-6-phosphate dehydrogenase (G6PDH) activity assay

RBCs were left untreated or treated with D-Gal, with or without pre-incubation with peel or juice extract. Glucose-6-phosphate dehydrogenase (G6PDH) activity was assessed using a commercial G6PDH activity assay kit (Sigma-Aldrich, Milan, Italy), according to the manufacturer’s instructions. The fluorescence intensity is proportional to the G6PDH activity in the samples. Determination of the reaction rate was performed by a plate spectrophotometer (Onda Spectrophotometer, UV-21) to monitor NAPDH rate of production, such molecule absorbs light at 340 nm, over a 30 min time course. Then the obtained reaction rate, presented as %, was normalized to total protein content by spectrophotometrically analysis at 540 nm wavelength needed to detect haemoglobin absorbance.

#### 2.12.4 Reduced glutathione (GSH) content measurement

GSH levels were quantified according to Teti and collaborators (Teti et al, 2005). Blood samples (20% hematocrit), which were left untreated or exposed to D-Gal with or without pre-incubation, were centrifuged (Neya 16R, 4°C, 1,200× g, 5 min) and resuspended in isotonic solution. After treatments, the content of GSH was measured by Cayman’s GSH assay kit using an enzymatic recycling method with glutathione reductase. Sample absorbance was measured at 412 nm (Onda spectrophotometer, UV-21). The amount of GSSG was calculated by the following formula: 1/2 GSSG = GSH total-GSH reduced. Results are expressed as a GSH/GSSG ratio.

### 2.13 Analysis and statistics

All data are expressed as arithmetic means ± standard error of the mean. GraphPad Prism (version 9.0, GraphPad Software, San Diego, CA, United States) and Excel (Version 2021, Microsoft, Redmond, WA, United States) software were used to perform statistical analysis and graphics. One-way analysis of variance (ANOVA) followed by Bonferroni’s multiple comparison or Dunnet’s post-test as appropriate, unless otherwise specified, was used to determine significant differences between mean values. Statistically significant differences between data sets were assumed at *p* < 0.05; (n) corresponds to the number of independent measurements.

## 3 Results

### 3.1 Determination of bioactive compounds

Table 1 reports qualitative and quantitative features of bioactive molecules deriving from bergamot juice and peel samples. In particular, thanks to the RP-HPLC/PDA/MS analyses, 26 bioactive molecules, of which three are phenolic acids, six limonoids and seventeen flavonoids, were identified and quantified. As seen in Table 1, the two sample sources showed the same qualitative profile.

**TABLE 1:** Concentration (mg Kg^−1^ ± standard deviation) of bioactive molecules in Femminello bergamot juice and peel. Each sample was analyzed in triplicate.

n°	Compound	Class^a^	Juice	Peel
1	Ferulic acid 4-O-glucoside^b^	PA	<LoD^c^	74.3 ± 0.27
2	Sinapoyl glucoside^d^	PA	<LoD^c^	208.8 ± 1.40
3	Apigenin 6,8-di-C-β-D-glucoside	F	4.2 ± 0.01	63.4 ± 0.80
4	Diosmetin-6,8-di-C-glucoside	F	3.9 ± 0.02	83.9 ± 0.78
5	Eriocitrin	F	16.0 ± 0.21	303.1 ± 3.38
6	Limonin glucoside	L	<LoD^c^	10.7 ± 0.52
7	Neoeriocitrin	F	15.5 ± 0.04	947.3 ± 4.97
8	5-Sinapoyquinic acid^d^	PA	<LoD^c^	19.7 ± 0.94
9	Poncirin^e^	F	24.9 ± 0.07	1811.6 ± 5.32
10	Diosmetin 8-C-glucoside^f^	F	<LoD^c^	155.4 ± 0.05
11	Narirutin	F	61.2 ± 0.21	853.4 ± 6.60
12	Naringin	F	12.2 ± 0.05	383.6 ± 2.93
13	Apigenin 7-O-neohesperidoside^e^	F	2.0 ± 0.05	65.6 ± 1.40
14	Deacetyl nomilin glucoside^g^	L	<LoD^c^	793.4 ± 1.94
15	Neodiosmin^e^	F	14.1 ± 0.46	1,533.9 ± 1.33
16	Apigenin 7-O-neohesperidoside-4-glucoside^e^	F	<LoD^c^	52.5 ± 1.22
17	Neohesperidin	F	300.0 ± 3.72	6,410.6 ± 11.04
18	Nomilin glucoside	F	<LoD^c^	964.4 ± 0.58
19	Nomilinic acid glucoside	F	<LoD^c^	445.4 ± 0.16
20	Apigenin 7-O-diglucuronide^e^	F	<LoD^c^	13.5 ± 0.34
21	Melitidin	F	10.4 ± 0.07	154.5 ± 2.08
22	Brutieridin	F	33.3 ± 0.09	494.4 ± 1.11
23	Ichangin^h^	L	<LoD^c^	39.3 ± 0.55
24	Obacunoic acid^h^	L	<LoD^c^	33.7 ± 0.22
25	Limonin	L	<LoD^c^	73.9 ± 0.03
26	Nomilin	L	<LoD^c^	123.8 ± 0.03
	All		498.1	16,132.3

Bioactive molecules were quantitatively determined based on calibration curves obtained with the corresponding standard compound, i.e.

^a^ PA, phenolic acid; F, flavonoid; L, limonoid.

^b^ ferulic acid.

^c^ LoD values ranged from 0.0.04 mg kg^−^–1.10 mg kg^−1^.

^d^ synapic acid.

^e^ apigenin 6,8-di-C-glucoside.

^f^ diosmetin 6,8-di-C-glucoside.

^g^ nomilin glucoside.

^h^ limonin. (Russo et al, 2015; Russo et al, 2016; Russo et al, 2021).

On the other hand, juice and peels differed from a quantitative point of view. Peel sample was richer in bioactive molecules (16,132.3 mg kg^−1^) than juice sample (498.1 mg kg^−1^). For both sources, the most abundant class of bioactive molecules (88%–94% of the total content) was represented by flavonoids. Neohesperidin was the most abundant bioactive compound in these samples.

### 3.2 Antioxidant capacity estimation of peel and juice extract

A series of experiments were conducted with increasing concentrations (1–250 μg/mL) of peel or juice extracts and incubation times (15 min-3 h) to exclude a possible hemolytic and pro-oxidant effect and estimate the antioxidant capacity of the peel or juice extract. Incubation with 1–5 μg/mL of peel or juice extract for 15 min at 37°C failed to induce hemolysis, increase TBARS levels, and reduce SH group content in RBCs (Supplementary Figures S1, S2). As expected, incubation of RBCs with 100 mM D-Gal for 24 h at 25°C led to a substantial increase in ROS and TBARS levels as well as a reduction in -SH group content compared to untreated RBCs (Supplementary Figure S3), which denotes induction of oxidative stress and is consistent our former findings (Remigante et al, 2022c). However, pre-incubation with 1 μg/mL of peel or juice extract for 15 min at 37°C did not significantly reduced ROS levels in D-Gal treated RBCs, while leading to minor effects on TBARS levels and -SH group content (Supplementary Figures S3A–C). Increasing concentration and pre-incubation times revealed a clear antioxidant effect of the peel and juice extracts, as denoted by a significant reduction in ROS and TBARS levels, as well as increase in total SH group content compared to D-Gal treated RBCs (Supplementary Figures S3D–I). Based on these data, we selected the most effective antioxidant concentration as well as the shortest effective time of incubation and pre-treatment with 5 μg/mL of peel or juice extract for 15 min has been chosen to carry out the following experiments.

#### 3.2.1 Evaluation of intracellular ROS levels

**FIGURE 1:**
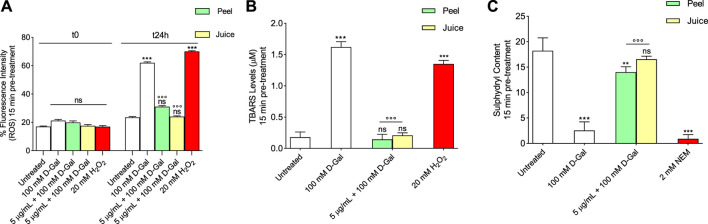
Antioxidant capacity estimation of peel and juice extract. human RBCs have been left untreated or exposed to 20 mM H_2_O_2_ or 2 mM NEM for 1 h or 100 mM D-Gal for 24 h at 25°C with or without pre-exposure to peel or juice extract for 15 min at 37°C. **(A)** estimation of ROS levels; **(B)** estimation of TBARS levels; **(C)** estimation of total sulfhydryl group content (µM TNB/µg protein). ns, not statistically significant *versus* control (untreated); **p < 0.01 and ***p < 0.001 *versus* control, ◦◦◦ *p* < 0.001 *versus* 100 mM D-Gal, ANOVA followed by Bonferroni’s multiple comparison post-test (*n* = 12).

Reactive oxygen species were detected in RBCs left untreated or, alternatively, exposed to 100 mM D-Gal for 24 h at 25°C with or without pre-exposure to 5 μg/mL peel or juice extract for 15 min at 37°C. Figure 1A displays the intracellular ROS levels at 0 and 24 h after exposure to D-Gal. As seen, 100 mM D-Gal treated samples showed a significant increase of ROS levels compared to left untreated samples. In samples pre-exposed to 5 μg/mL peel or juice extract, the incubation with 100 mM D-Gal failed to significantly increase ROS levels, which remained unchanged when compared to control values (Figure 1A). As expected, ROS levels of RBCs treated with 20 mM H_2_O_2_ for 30 min were significantly higher with respect to those of RBCs left untreated (control). In addition, peel or juice extract alone did not significantly alter the intracellular ROS levels (data not shown).

#### 3.2.2 Measurement of thiobarbituric acid reactive substances (TBARS) levels

Thiobarbituric-acid-reactive substances (TBARS) levels measured in RBCs are reported in Figure 1B. As expected, TBARS levels after treatment with 20 mM H_2_O_2_ for 1 h were significantly higher than those detected in control (left untreated RBCs). Similarly, after 24 h treatment with 100 mM D-Gal, TBARS levels were significantly increased with respect to control. Importantly, in RBCs pre-treated with 5 μg/mL of peel or juice extract and then exposed to 100 mM D-Gal, TBARS levels were significantly lower than those measured in 100 mM D-Gal-treated RBCs. Of note, peel or juice extract alone did not significantly alter TBARS levels (Supplementary Figures S1D, S2D).

#### 3.2.3 Total sulfhydryl group content measurement

Figure 1C shows the total content of sulfhydryl groups in left untreated RBCs or treated with either the oxidizing molecule NEM as positive control (2 mM for 1 h) or d-Gal (100 mM for 24 h) with or without pre-treatment with peel or juice extract (5 μg/mL). As expected, treatment with NEM led to a significant reduction in the content of the sulfhydryl groups compared to untreated RBCs (control). Sulfhydryl groups in - RBCs treated with 100 mM D-Gal were also significantly lower than control. The total content of the sulfhydryl groups in 100 mM D-Gal-treated RBCs was significantly restored in case of pre-treatment with peel or juice extract (5 μg/mL) (Figure 1C). Additionally, peel or juice extract alone did not significantly alter the total content of the sulfhydryl groups (Supplementary Figures S1G, S2G).

### 3.3 Determination of band 3 expression levels

**FIGURE 2:**
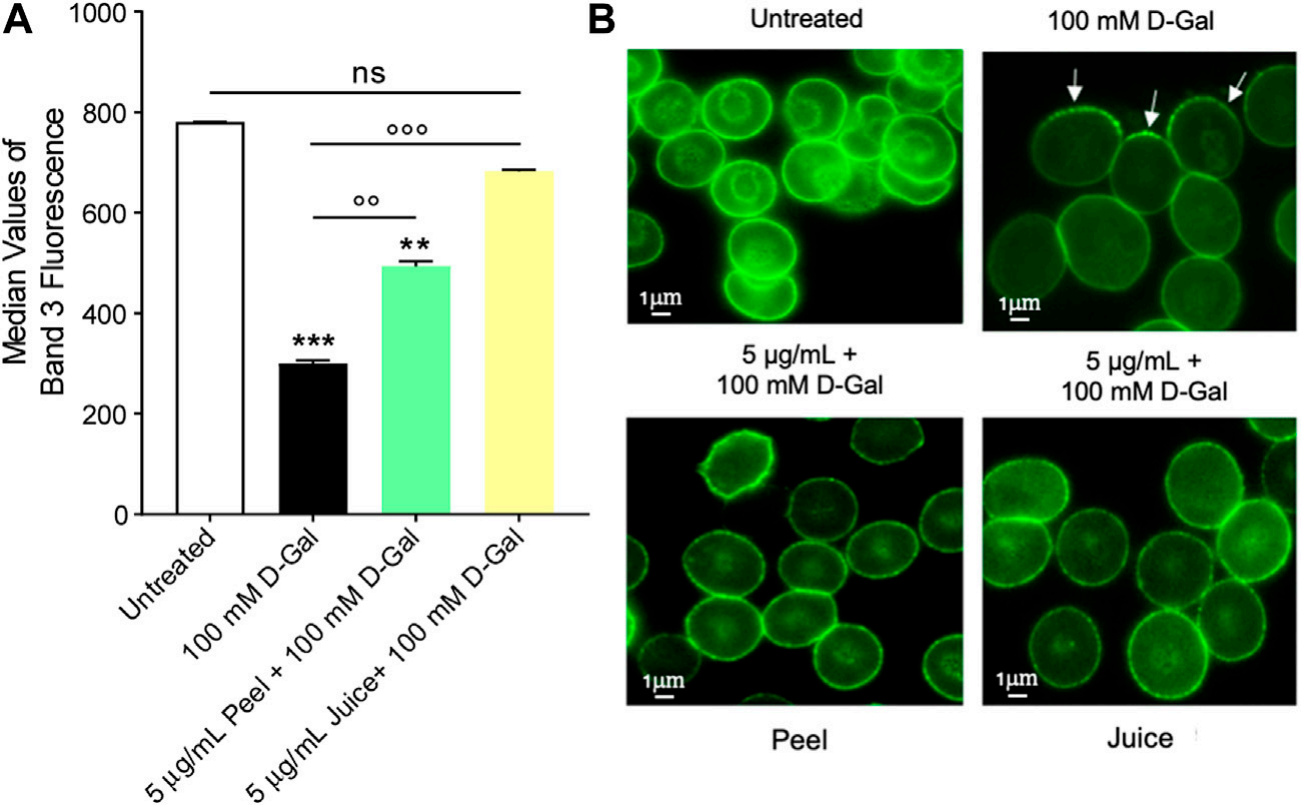
Flow cytometry immunofluorescence of band 3 expression. Red blood cells were left untreated or treated with 100 mM D-Gal for 24 h at 25°C, with or without pre-exposure to 5 μg/mL peel or juice extract for 15 min at 37°C. **(A)** Histograms report median values of fluorescence intensity. **(B)** Flow cytometry immunofluorescence representative micrographs showing band 3 distribution in left untreated RBCs, treated with 100 D-Gal, or alternatively, pre-incubated with 5 μg/mL peel or juice extract, and then exposed to 100 mM D-Gal. Samples were observed with a ×100 objective. Note the significant morphological changes in 100 mM D-Gal (arrows). ns, not statistically significant *versus* control (untreated); **p < 0.01 and ***p < 0.001 *versus* control, ◦◦ *p* < 0.01 and ◦◦◦ *p* < 0.001 *versus* 100 mM D-Gal, ANOVA followed by Bonferroni’s multiple comparison post-test (*n* = 3).

The levels of band 3 expression were significantly decreased in human RBCs incubated with 100 mM D-Gal for 24 h with respect to control (Figure 2A). Pre-treatment with 5 μg/mL peel extract partially restored band 3 expression in RBCs treated with 100 Mm D-Gal. Also, band 3 expression was totally restored in RBCs pre-exposed to 5 μg/mL juice extract. Moreover, Band 3 distribution was assessed by immunofluorescence technique. In particular, band 3 was mainly clustered (arrows) in leptocytes after 100 mM D-Gal exposure, with respect to untreated RBCs (Figure 2B). These changes were attenuated by pre-treatment with 5 μg/mL or juice extract. Of note, band 3 expression was altered neither by peel nor by juice extracts given alone (data not shown).

### 3.4 SO_4_^2−^ uptake measurement

**FIGURE 3:**
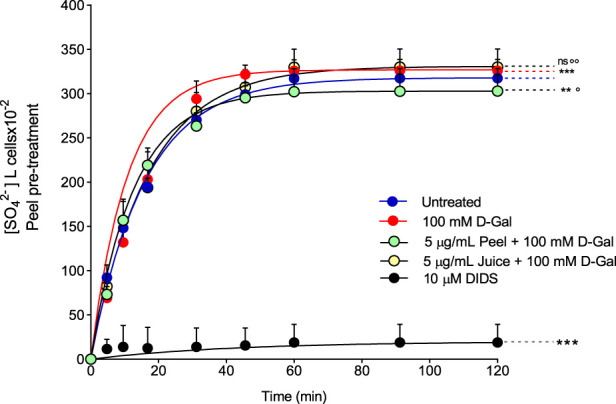
Time course of SO_4_^2−^ uptake. Red blood cells were left untreated (control) or treated with 100 mM D-Gal (24 h, at 25°C) with or without pre-incubation with 5 μg/mL peel or juice extract (15 min at 37°C). Red blood cells were also exposed to a specific inhibitor of band 3 (10 µM DIDS). ns, not statistically significant *versus* untreated (control); **p < 0.01 and ***p < 0.001 *versus* control; ◦ *p* < 0.05 and ◦◦ *p* < 0.01 *versus* 100 mM D-Gal, one-way ANOVA followed by Bonferroni’s multiple comparison *post hoc* test (*n* = 15).

To evaluate the band 3 activity, the SO42− uptake during the time was determined in RBCs left untreated (control) or treated with 100 mM d-Gal for 24 h at 25°C, with or without pre-exposure to 5 μg/mL peel or juice extract for 15 min at 37°C (Figure 3). In left untreated RBCs, SO42− uptake is seen to progressively increase reaching equilibrium in 16.68 min (rate constant of SO42− uptake = 0.059 ± 0.001 min−1, Table 2). The transport rate constant in RBCs treated with 100 mM d-Gal (0.092 ± 0.001 min−1) was significantly increased with respect to control, thus denoting an accelerated transport kinetics, which was in agreement with former findings (Remigante et al, 2022c). Pre-exposure to 5 μg/mL peel or juice extract significantly reduced the rate constant of SO42− uptake, which did not differ from control in the case of pre-treatment with peel extract. The SO42− amount internalized by RBCs after 45 min of incubation in SO42− medium was not significantly altered by d-Gal with or without peel or juice extract. In DIDS-treated cells, rate constant of SO42− uptake and the SO42− amount internalized were substantially reduced compared to control, consistent with a SO42− transport via band 3 (Table 2). Red blood cells treated with 5 μg/mL peel or juice extract alone showed a time course of SO42−uptake that was not significantly altered compared to control (Supplementary Figure S4).

**TABLE 2:** Rate constant of SO_4_^2−^ uptake, time needed to reach equilibrium, and SO_4_^2−^ quantity internalized by either RBCs untreated or treated as indicated. Data are presented as means ± S.E.M. from (n) separate experiments. ns not statistically significant *versus* untreated (control); ***p < 0.001 *versus* untreated; ° *p* < 0.01 *versus* 100 mM D-Gal, as attested by one-way ANOVA followed by Bonferroni’s multiple comparison *post hoc* test.

Experimental conditions	Rate constant (min^−1^)	Time (min)	n	SO_4_^2−^ amount trapped after 45 min of incubation in SO_4_^2−^ medium (SO_4_^2−^) l cells X 10^–2^
Untreated	0.059 ± 0.001	16.68	15	299.08 ± 9.95
100 mM D-Gal	0.092 ± 0.001^***^	10.74	15	321.75 ± 10.57^ns^
5 μg/mL Peel Extract +100 mM D-Gal	0.059 ± 0.001 ^ns,°^	16.70	15	307.50 ± 19.75^ns^
5 μg/mL Juice Extract +100 mM D-Gal	0.079 ± 0.001^ns,°^	12.52	15	295.25 ± 10.37^ns^
10 µM DIDS	0.023 ± 0.001^***^	42.17	15	15.5 ± 0.37^***^

### 3.5 Advanced glycation end products (AGEs): measurement of glycated haemoglobin (A1c) levels

**FIGURE 4:**
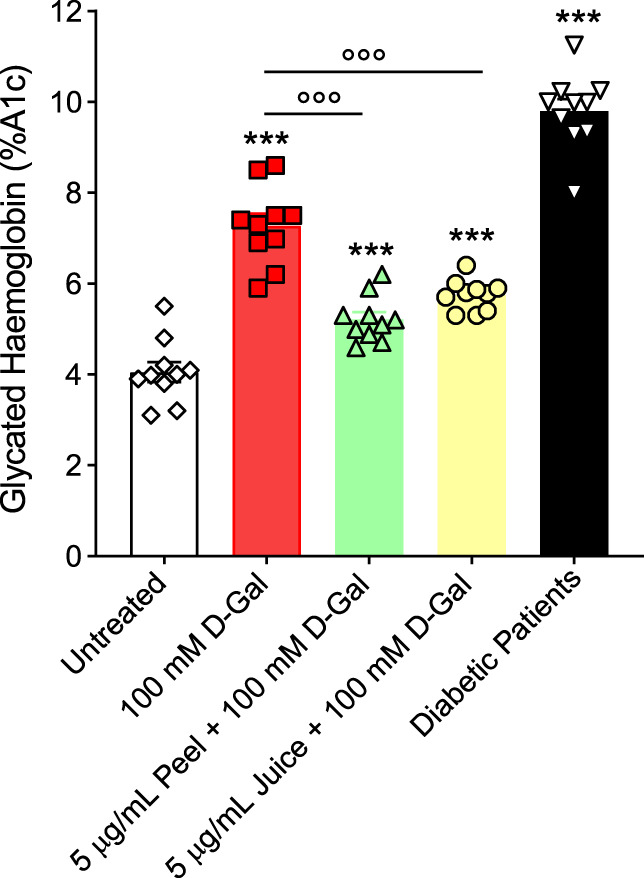
Glycated haemoglobin levels (%A1c). Red blood cells were left untreated or treated with 100 mM D-Gal (24 h, at 25°C) with or without pre-incubation with 5 μg/mL peel or juice extract (15 min at 37°C). ns, not statistically significant *versus* untreated; ***p < 0.001 *versus* untreated; ◦◦◦ *p* < 0.01 *versus* 100 mM D-Gal, one-way ANOVA followed by Bonferroni’s multiple comparison *post hoc* test (*n* = 10).

Figure 4 displays the % A1c determined in left untreated RBCs or treated with 100 mM d-Gal for 24 h, with or without pre-exposure to 5 μg/mL peel or juice extract for 15 min at 37°C. The data obtained showed that %A1c levels measured after exposure to 100 mM d-Gal were significantly higher than those of RBCs left untreated (control). Instead, in RBCs pre-treated with 5 μg/mL peel or juice extract, a trend towards a decrease in %A1c levels was seen, which was statistically significant although %A1c levels remained elevated compared to control. In Figure 4, A1c levels measured in diabetes patients -used as the positive control-are also reported (Chandalia and Krishnaswamy, 2002). As expected, a significant increase in the A1c levels were measured in RBCs from diabetic patients. Lastly, %A1c content was not significantly altered by peel or juice extract when applied alone (data not shown).

### 3.6 Assessment of the endogenous antioxidant activity

#### 3.6.1 Activity of superoxide dismutase (SOD)

**FIGURE 5:**
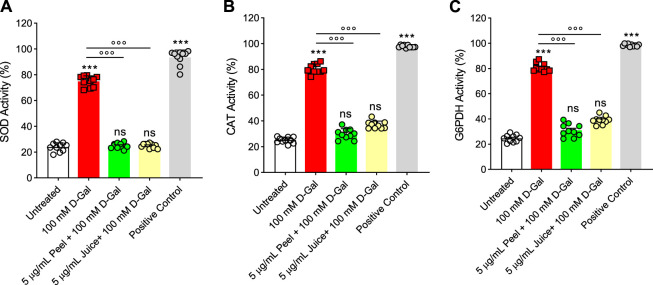
Effects of D-Gal (100 mM) exposure with or without pre-treatment with peel or juice extract for 15 min in RBCs. **(A)** SOD activity, **(B)** CAT activity, and **(C)** G6PDH activity. ns, not significant *versus* untreated (control); ***p < 0.001 *versus* untreated; ◦◦◦*p* < 0.001 *versus* 100 mM D-Gal, as determined by one-way ANOVA followed by Bonferroni’s multiple comparison *post hoc* test (*n* = 10).

In Figure 5A, the SOD activity was evaluated in RBCs left untreated or treated with D-Gal (100 mM for 24 h at 25°C) with or without pre-exposure to 5 μg/mL peel or juice extract for 15 min at 37°C. In RBCs exposed to D-Gal, the SOD activity was found significantly increased compared to the untreated cells. Conversely, pre-incubation with peel or juice extract resulted in a significant recovery of SOD activity with respect to D-Gal-treated cells. As expected, SOD activity in RBCs treated with 20 mM H2O2 for 30 min at 25°C as the positive control was significantly higher than that of control RBCs. Of note, peel and juice extracts did not significantly alter SOD activity when given alone (data not shown).

#### 3.6.2 Activity of catalase (CAT)

Catalase was assayed in RBCs left untreated or treated with D-Gal (100 mM for 24 h at 25°C), with or without pre-treatment with 5 μg/mL peel or juice extract (15 min at 37°C). D-Gal treatment caused an increased CAT activity compared to control cells, which was consistent with an elevated oxidative stress (Figure 5B). Unlike, the pre-incubation with peel and/or juice extract (5 μg/mL for 15 min at 37°C) showed a CAT activity almost comparable to that of control (Figure 5B). Exposure to 20 mM H_2_O_2_ for 30 min (25°C) has been considered as the positive control. As expected, CAT activity in RBCs treated with H_2_O_2_ was significantly higher than those of control RBCs. Moreover, the extracts of peel and juice alone did not significantly alter CAT activity (data not shown).

#### 3.6.3 Activity of glucose-6-phosphate dehydrogenase (G6PDH)

In Figure 5C, G6PDH activity was measured in RBC left untreated or treated with D-Gal (100 mM for 24 at 25°C), with or without pretreatment with 5 μg/mL peel or juice extract (15 min at 37°C). G6PDH activity was severely stimulated by an increased oxidative stress in the D-Gal-treated cells compared to control RBCs. On the contrary, in cells pre-exposed to peel or juice extract, G6PDH activity was brought back to the control levels. As expected, G6PDH activity of RBCs treated with G6PDH positive control supplied by the manufacturer for 30 min at 25°C was significantly higher than that of RBCs left untreated. In addition, both peel and juice extracts did not significantly alter G6PDH activity (data not shown).

#### 3.6.4 Estimation of GSH/GSSG ratio

**FIGURE 6:**
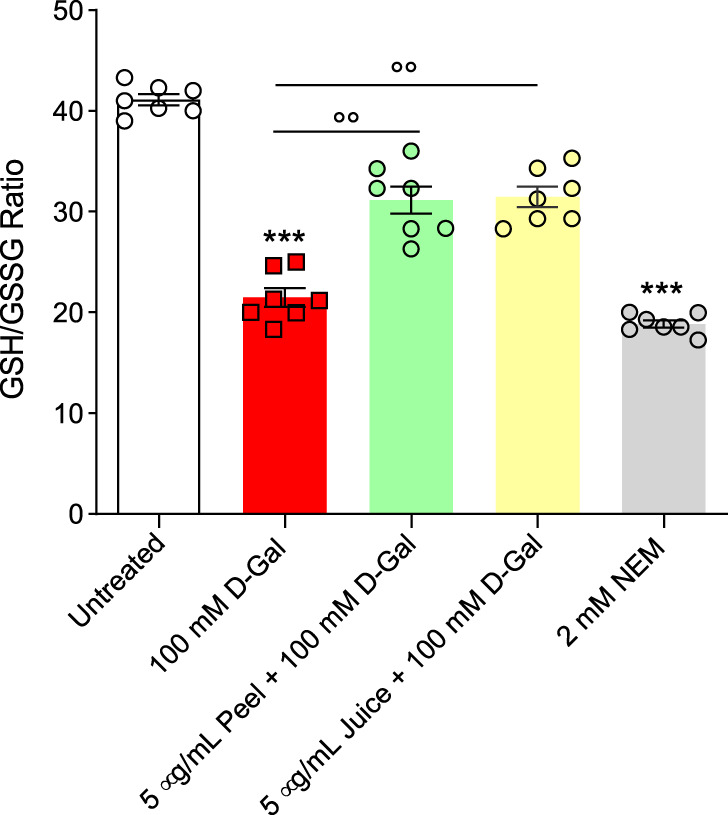
Assessment of the GSH/GSSG ratio measured in RBCs incubated for 24 h at 25°C with D-Gal with or without pre-treatment with peel or juice extract (15 min at 37°C). GSH, reduced glutathione; GSSG, oxidized glutathione. ***p < 0.001 *versus* left untreated RBCs; ◦◦*p* < 0.01 *versus* D-Gal, one-way ANOVA followed by Bonferroni’s multiple comparison *post hoc* test (*n* = 7).

Figure 6 displays the GSH/GSSG ratio measured in RBCs treated with 100 mM D-Gal for 24 h at 25°C with or without pre-treatment with 5 μg/mL peel and juice extract for 15 min at 37°C. The GSH/GSSG ratio measured after treatment with D-Gal was significantly lower than that detected in left untreated RBCs. This effect could be associated with an increased GSSG levels and/or decreased GSH levels, both of which are indicative of an increased intracellular oxidative stress. However, pre-incubation with peel and juice extract restored GSH levels. As expected, incubation with 2 mM NEM for 1 h (used as the positive control) led to a significant reduction in the GSH/GSSG content compared to left untreated RBCs. On the contrary, both peel and juice extract alone did not significantly alter the GSH/GSSG content (data not shown).

## 4 Discussion

Recently, an increasing body of evidence has supported the hypothesis that natural molecules -referred to as antioxidants-may have a protective role in retarding or reversing the course of age-related diseases (Tan et al, 2018; Singh et al, 2020; Lu et al, 2021; Remigante et al, 2022b; Cui et al, 2022). These compounds are able to compete with substrates which are sensitive to oxidation, thus inhibiting or delaying the reactions between reactive species and biological macromolecules (Pisoschi and Pop, 2015). Even though reactive species are involved in several biological processes, their over-production can lead to cell damage and consequently, development of chronic diseases (Costa et al, 2020; Luo et al, 2020; Bertelli et al, 2021; Remigante et al, 2021b; Forman and Zhang, 2021; Zuccolini et al, 2022). Thus, dietary intake of natural antioxidant compounds could work as a boost to the endogenous antioxidant machinery against reactive species and/or free radicals, thus playing an important role in the prevention of pathological states (Pingitore et al, 2015; Gantenbein and Kanaka-Gantenbein, 2021). Herein, the composition in bioactive molecules of bergamot peel and juice (Table 1) and their effects on oxidative-stress induced aging were described in a cell-based model represented by human RBCs subjected to prolonged (24 h) exposure to 100 mM D-Gal. This cell-based model, which was validated in our former studies (Remigante et al, 2022c; Remigante et al, 2022d; Spinelli et al, 2023), recapitulates the cellular and molecular mechanisms of natural aging, i.e., oxidative stress and haemoglobin glycation.

Though various investigations described the numerous activities of bioactive compounds of bergamot extracts, their effects on aging RBCs have not yet been fully analysed. Then, the first step of the present investigation was to test a broad range of concentrations (from 1 μg/mL to 250 μg/mL) of bergamot peel and juice extract, as well as increasing incubation time intervals (15 min, 1 and 3 h), to exclude any damage in terms of haemolysis, lipid peroxidation and protein oxidation that could potentially be caused by direct exposure of RBCs to peel or juice extracts (Supplementary Figures S1, S2). Indeed, concentrations of peel or juice extract equal to or greater than 10 μg/mL could induce haemolysis and exhibited a pro-oxidant effect, especially following prolonged incubation times. This is not surprising, given that various antioxidants may act as pro-oxidants depending on their concentration (Osseni et al, 2000; Sahebkar, 2015; Giordano et al, 2020). These and our findings point to the importance of carefully assessing concentration and incubation time when novel potential antioxidant compounds are tested in cell-based assays. Based on these considerations, we selected a 15 min pre-treatment with 5 μg/mL peel or juice extract in order to estimate the antioxidant capacity by measuring ROS and TBARS levels, and total content of sulfhydryl groups in RBCs incubated for 24 h with 100 mM D-Gal (Figure 1).

Red blood cells are susceptible to ROS-induced damage because of their high polyunsaturated fatty acid content and their abundance in iron (Fe^2+^)-rich haemoglobin. This latter acts as a catalyst in redox reactions and lipid peroxidation, resulting in TBARS production as the final product (Pandey and Rizvi, 2010). Also, RBCs often undergo membrane protein oxidation and/or carbonylation. Therefore, the oxidation of protein sulfhydryl groups (-SH) and/or the formation of carbonyl groups are indicators of oxidative injury to proteins in human RBCs. Since ROS generated during cellular metabolism cause the oxidation of biological macromolecules, the effect of peel and juice extract on the intracellular ROS levels has been evaluated as a first step of the investigation. A 15 min pre-exposure of RBCs to 5 μg/mL peel or juice extract could effectively prevent the ROS production caused by exposure to D-Gal (Figure 1A).

To better elucidate the process of oxidation of plasma membrane macromolecules, the estimation of both TBARS level and sulfhydryl group content of total proteins, which in RBCs mainly belong to band 3 protein (Rao and Reithmeier, 1979), have been investigated. Our findings show that pre-exposure (15 min) to 5 μg/mL peel or juice extract completely prevented TBARS levels increase in RBCs treated with 100 D-Gal (Figure 1B) and could at least partially (peel) or completely (juice) restore sulfhydryl group content (Figure 1C). These findings denote that peel or juice extract could effectively protect both the lipid and protein component of the RBC plasma membrane from oxidation. Data related to oxidative stress assessment are in line with what previously showed by other researchers and suggest that polyphenols and phytochemicals could play scavenger activity by directly neutralizing reactive species and free radicals and avoid their detrimental impact on biological macromolecules (Zhao et al, 2006; Kelen and Tepe, 2007; Luo et al, 2021; Kruk et al, 2022).

One of the most interesting and still unknown implications of oxidative stress-related aging is its impact on plasma membrane transport systems. In this regard, several investigations demonstrated that the aging process may impact the anion (Cl^−^/HCO_3_^−^) exchange, mediated by band 3, on the RBC plasma membrane. The structure of this protein consists of two rather different domains of a similar size (Arakawa et al, 2015). The C-terminal domain carries out Cl^−^/HCO_3_^−^ exchange across the plasma membrane (Reithmeier et al, 2016; Remigante et al, 2022e). This function can be monitored by the rate constant for sulphate (SO_4_^2−^) uptake (Morabito et al, 2016; Morabito et al, 2017a; Morabito et al, 2017b), which is slower and more easily detectable than Cl^−^ or HCO_3_^−^ uptake (Jennings, 1976; Romano and Passow, 1984; Morabito et al, 2019b; Crupi et al, 2019). SO_4_^2−^ uptake measurement has been firmly confirmed as an efficient tool to investigate the impact of redox conditions on RBCs homeostasis (Morabito et al, 2016; Morabito et al, 2019b; Remigante et al, 2019). Hence, the SO_4_^2−^ uptake through band 3 was measured in RBCs after treatment with D-Gal (100 mM) with or without pre-exposure (15 min) to 5 μg/mL of bergamot peel or juice extract. In RBCs treated with D-Gal (100 mM), the rate constant for SO_4_^2−^ uptake was accelerated compared to the control (Figure 3; Table 2). The evidence that oxidative stress provokes functional modifications of band 3 in human RBCs has been provided also in other cell-based models of oxidative stress. In particular, acute H_2_O_2_-induced oxidative stress provoked a reduction in the rate constant for SO_4_^2−^ uptake (Morabito et al, 2016; Morabito et al, 2017b), whereas in an oxidative stress model induced by high glucose concentrations an accelerate rate of ì anion exchanging has been seen (Morabito et al, 2020b). Thus, a possible a two-faced effect on the velocity of anion exchange could depend on specific cell component (lipids, proteins, as well as enzymes) affected by both the stressors and the related pathways. In this context, although no functional alteration was reported in RBCs treated exclusively with 5 μg/mL peel or juice extract (Supplementary Figure S4), a 15 min pre-treatment of RBCs formerly exposed to D-Gal partially or totally recovered the rate constant for SO_4_^2−^ uptake (Figure 3; Table 2). Based on data hitherto obtained, we can point out that both extracts show a similar protective effect on anionic exchange and could, therefore, act a key role in counteracting oxidative stress–induced functional changes in human RBCs.

The binding sites for cytoskeletal and cytoplasmic proteins, including haemoglobin, are located on the N-terminal cytoplasmic domain of band 3 (Anong et al, 2009; Wu et al, 2011). Haemoglobin glycation represents a case of non-enzymatic protein glycation associated with production of AGEs (Luevano-Contreras and Chapman-Novakofski, 2010). To better explore the molecular interaction between band 3 and haemoglobin, levels of both proteins were evaluated. The data obtained show that 100 mM D-Gal treatment caused both a loss and a redistribution of band 3, most probably due to the shedding of protein-containing vesicles (Kuo et al, 2017) (Figures 2A, B). Yet, despite this, band 3 expression levels were partially or totally restored in RBCs pre-treated with 5 μg/mL peel or juice extract, respectively (Figures 2A, B). The shedding of membrane area is a critical point for cell fate, since a reduced surface-to-volume ratio is thought to correlate with the early phagocytosis of aged RBCs (Kuo et al, 2017). Consequently to membrane shedding, the cytoskeleton reduces to a 3- to 5-fold smaller area (Li and Lykotrafitis, 2015). The proteins affected are mainly band 3, ankyrin, spectrin, and occasionally protein 4.2 and the Rh protein. These proteins are all part of one of the complexes by which the cytoskeleton is anchored to the lipid bilayer (Gardel et al, 2008; Anong et al, 2009; Vallese et al, 2022). Presumably, damage of these proteins is responsible for the impaired cellular deformability associated with oxidative stress (Mohanty et al, 2014). A decrease in RBC deformability leads to impaired microcirculation and tissue oxygenation (Schwartz et al, 1991; Silva-Herdade et al, 2016). For example, Spinelli and co-authors have shown that both band 3 phosphorylation and rearrangements in cytoskeleton proteins can lead to cell deformation and structural alteration of human RBCs during the aging processes (Spinelli et al, 2023).

As mentioned above, haemoglobin is anchored to band 3 cytoplasmic domain. In this regard, our findings indicated that exposure of RBCs to 100 mM D-Gal for 24 h increased the content of A1c levels (Figure 4), contributing to both biochemical and structural changes, including clustering of band 3 regions. At a more advanced stage of aging, such clusters could represent a recognition site for antibodies directed against aged RBCs, thus triggering the early removal of RBCs from the blood circulation (Briglia et al, 2017). Importantly, in RBCs pre-treated with 5 μg/mL peel or juice extract, a clear trend towards a decrease in %A1c production was seen (Figure 4). Collectively, these findings (Figures 2, 4) suggest that the active biomolecules of the bergamot juice or peel extract might efficiently prevent RBCs membrane shedding, formation of glycated haemoglobin, and RBCs structural instability, all of which are hallmarks of aging.

At last, we investigated the activity of endogenous antioxidant enzymes CAT and SOD (Figures 5A, B). These antioxidant enzymes own outstanding free radical scavenging capacities and play vital roles in human RBCs (Ulanczyk et al, 2020). The SOD and CAT activity in RBCs incubated with 100 mM D-Gal was much higher than in control cells, which could reflect the activation of the endogenous antioxidant defense system to suppress the formation of free radicals (Figure 1A). Nevertheless, the increase in SOD and CAT activity failed to compensate for the increase in free radicals (Figure 1A), as also demonstrated by the increase in lipid peroxidation levels as well as total protein oxidation (Figures 1B, C). Upregulation in CAT and SOD activity with concomitant substantial elevation of oxidative stress markers might reflect exhaustion of the endogenous antioxidant system. These biochemical changes may provoke injury to the membrane proteins and lipid structure. As a result, the membrane mechanical properties could be modified, resulting in deformability and fluidity reduction or altered permeability of the phospholipid bilayer, which, in turn, reduce the ability of the membrane to withstand osmotic changes (Spinelli et al, 2023). In this context, pre-exposure of RBCs to peel and juice extracts could significantly prevent the upregulation in both SOD and CAT activity observed in D-Gal-treated cells (Figures 5A, B). These findings denote that the active biomolecules of the bergamot juice or peel extract might act synergistically with endogenous antioxidant system to counteract oxidative stress in RBCs and preserve cell integrity.

RBCs are unable to generate ATP using molecular oxygen because of lack of mitochondria. Glycolysis is the only source of ATP generation in mature RBCs. It has been found that under normal conditions about 90% of total imported glucose is used to generate ATP through glycolysis and the remaining 10% of glucose is addressed to PPP (Reisz et al, 2016). Glucose-6-phosphate dehydrogenase (G6PDH) is a crucial enzyme of this latter pathway, which produces reduced NADPH and represents the most important defense mechanism for RBCs in case of excessive oxidant generation, or of ineffective antioxidant defense (Maha, 2009). In these conditions, glycolysis and its key enzyme GAPDH are inhibited (Reisz et al, 2016). In addition to its importance in the RBC as a major metabolic determinant during oxidative stress, G6PDH activity is also employed as an indication of cell aging (Subasinghe and Spence, 2008). The G6PDH activity was severely stimulated - presumably by an increased oxidative stress - in D-Gal-treated cells compared to control RBC cells (Figure 5C), which was consistent with inhibition of glycolysis and activation of the PPP shunt. Vice versa, in cells pre-exposed to peel and/or juice extract the G6PDH activity was brought back to the control levels (Figure 5C). In addition, as already mentioned, the underlying mechanisms of aging can severely alter enzyme activities, including glutathione (Maher, 2005)**.** Glutathione is an important endogenous non-enzymatic antioxidant that neutralizes ROS production (Ahmad and Mahmood, 2019). Its depletion makes cells more prone to oxidative damage. Then, the GSH/GSSG ratio was assayed. The obtained results confirmed that 100 mM D-Gal treatment reduced the GSH/GSSG ratio. However, the pre-incubation of RBCs with peel and juice extract partially restored the redox balance (Figure 6). In summary, our findings suggest that the active biomolecules of the bergamot juice or peel extract might prevent metabolic alterations in RBCs exposed to oxidative stress.

## 5 Conclusion

Here we characterize the precise composition in bioactive compounds of the bergamot (Citrus bergamia, Femminello cultivar) peel and juice extracts and we assess their protective effect in an oxidative stress-related model of cellular aging in RBCs. The data presented here indicate, for the first time, that polyphenol-rich extracts of peel and juice from bergamot fruit may act on oxidative stress-induced alterations of the lipid and protein cellular components, including the endogenous antioxidant system as well as proteins with ion transport activity and enzymatic metabolic activity, thus protecting the structural and functional integrity of human RBCs. This study identifies bergamot as an antioxidant functional food and further suggests that diet supplementation with bergamot or its derivatives might contribute to the prevention or attenuation of pathophysiological events linked to RBCs dysfunction during aging.

## Data Availability

The raw data supporting the conclusion of this article will be made available by the authors, without undue reservation.
