# Supplementary material for: Mechanisms underlying the anti-aging activity of bergamot (Citrus bergamia) extract in human red blood cells
Source: Front Physiol. 2023 Jun 30;14:1225552. doi: 10.3389/fphys.2023.1225552 (PMC10348362; doi:10.3389/fphys.2023.1225552)
Supplement: Supplementary file 1 [file Presentation1.PPTX]

## Slide 1
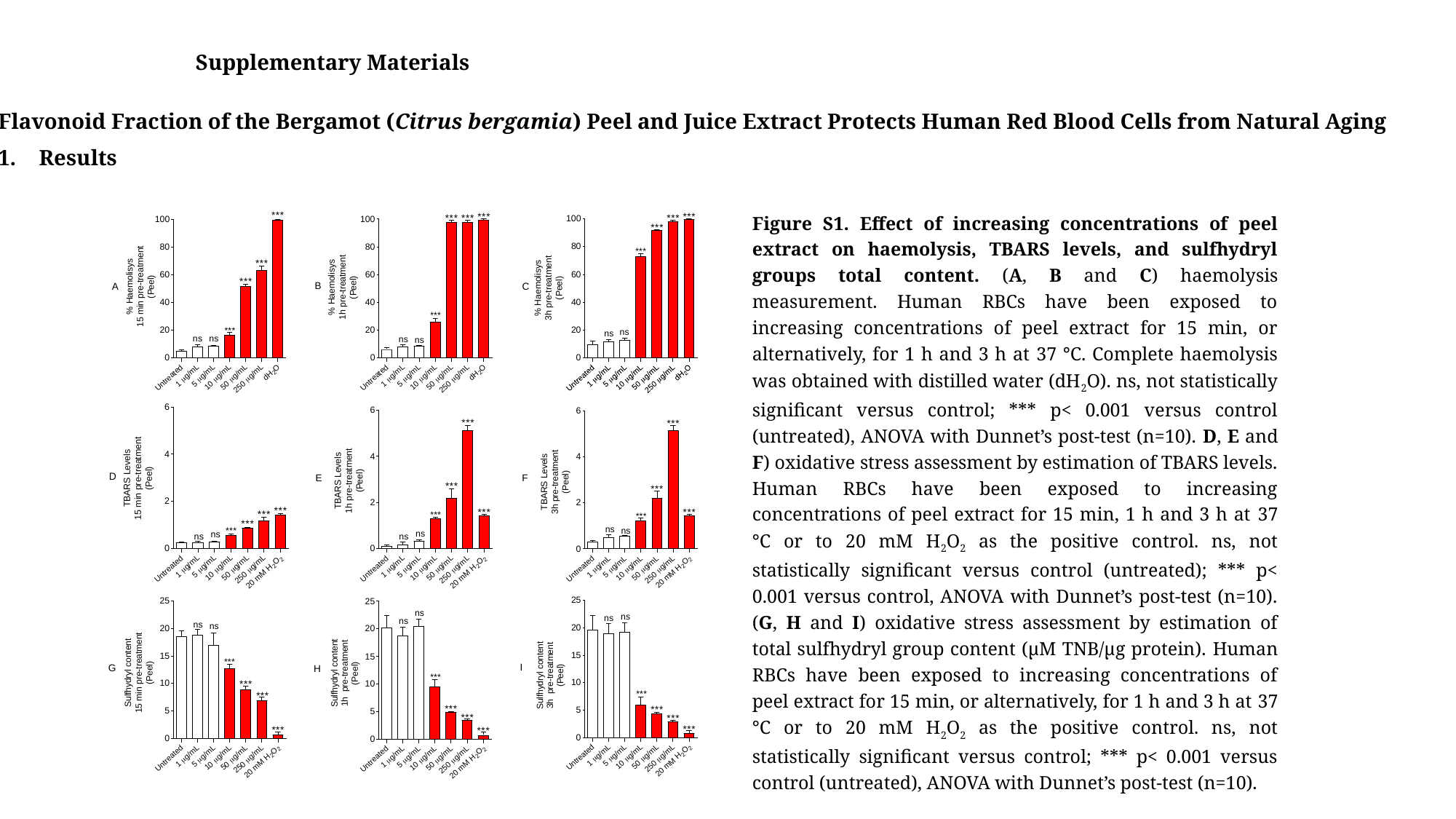

Supplementary Materials
Flavonoid Fraction of the Bergamot (Citrus bergamia) Peel and Juice Extract Protects Human Red Blood Cells from Natural Aging
Results
Figure S1. Effect of increasing concentrations of peel extract on haemolysis, TBARS levels, and sulfhydryl groups total content. (A, B and C) haemolysis measurement. Human RBCs have been exposed to increasing concentrations of peel extract for 15 min, or alternatively, for 1 h and 3 h at 37 °C. Complete haemolysis was obtained with distilled water (dH2O). ns, not statistically significant versus control; *** p< 0.001 versus control (untreated), ANOVA with Dunnet’s post-test (n=10). D, E and F) oxidative stress assessment by estimation of TBARS levels. Human RBCs have been exposed to increasing concentrations of peel extract for 15 min, 1 h and 3 h at 37 °C or to 20 mM H2O2 as the positive control. ns, not statistically significant versus control (untreated); *** p< 0.001 versus control, ANOVA with Dunnet’s post-test (n=10). (G, H and I) oxidative stress assessment by estimation of total sulfhydryl group content (µM TNB/µg protein). Human RBCs have been exposed to increasing concentrations of peel extract for 15 min, or alternatively, for 1 h and 3 h at 37 °C or to 20 mM H2O2 as the positive control. ns, not statistically significant versus control; *** p< 0.001 versus control (untreated), ANOVA with Dunnet’s post-test (n=10).

## Slide 2
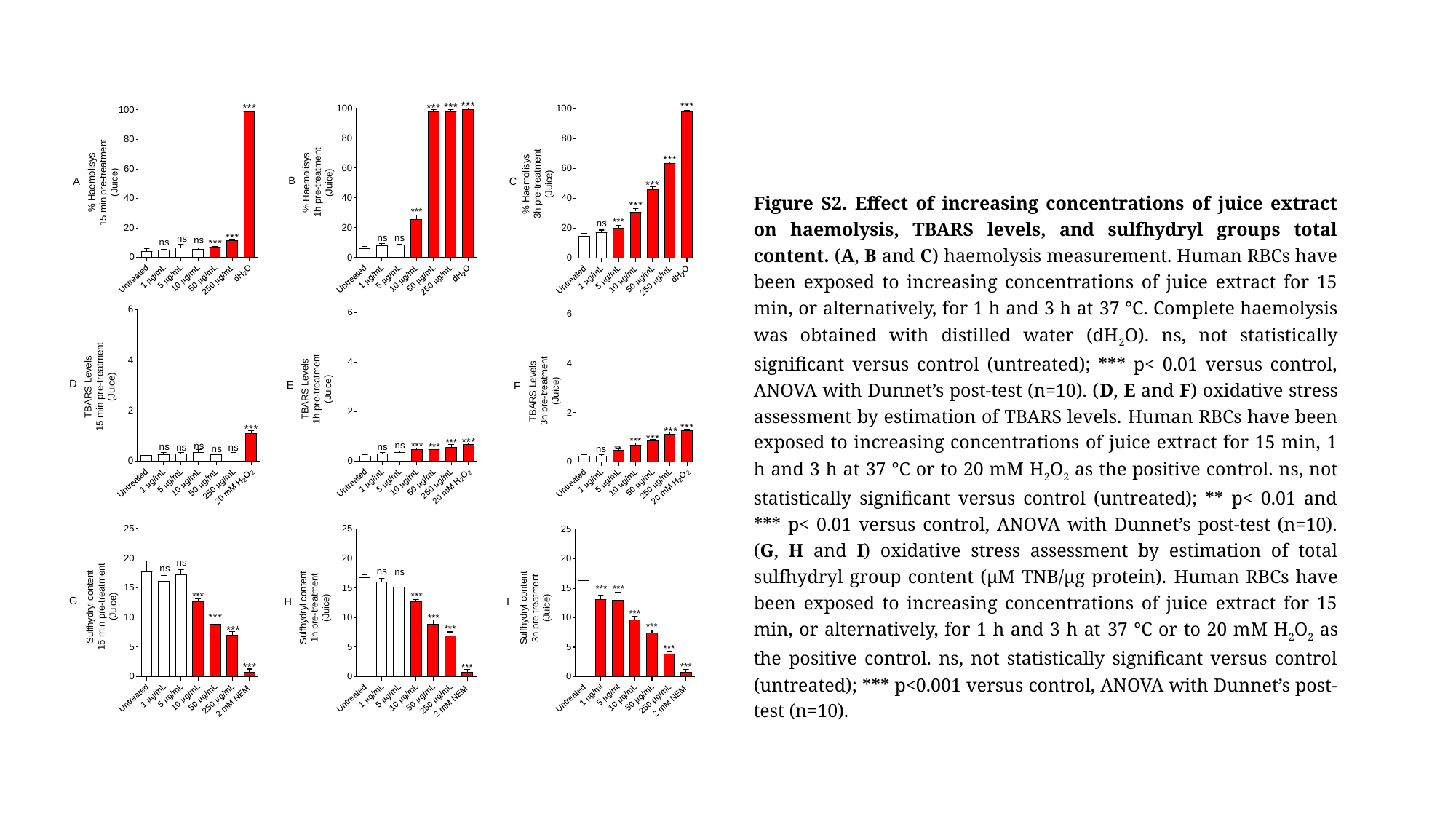

Figure S2. Effect of increasing concentrations of juice extract on haemolysis, TBARS levels, and sulfhydryl groups total content. (A, B and C) haemolysis measurement. Human RBCs have been exposed to increasing concentrations of juice extract for 15 min, or alternatively, for 1 h and 3 h at 37 °C. Complete haemolysis was obtained with distilled water (dH2O). ns, not statistically significant versus control (untreated); *** p< 0.01 versus control, ANOVA with Dunnet’s post-test (n=10). (D, E and F) oxidative stress assessment by estimation of TBARS levels. Human RBCs have been exposed to increasing concentrations of juice extract for 15 min, 1 h and 3 h at 37 °C or to 20 mM H2O2 as the positive control. ns, not statistically significant versus control (untreated); ** p< 0.01 and *** p< 0.01 versus control, ANOVA with Dunnet’s post-test (n=10). (G, H and I) oxidative stress assessment by estimation of total sulfhydryl group content (µM TNB/µg protein). Human RBCs have been exposed to increasing concentrations of juice extract for 15 min, or alternatively, for 1 h and 3 h at 37 °C or to 20 mM H2O2 as the positive control. ns, not statistically significant versus control (untreated); *** p<0.001 versus control, ANOVA with Dunnet’s post-test (n=10).

## Slide 3
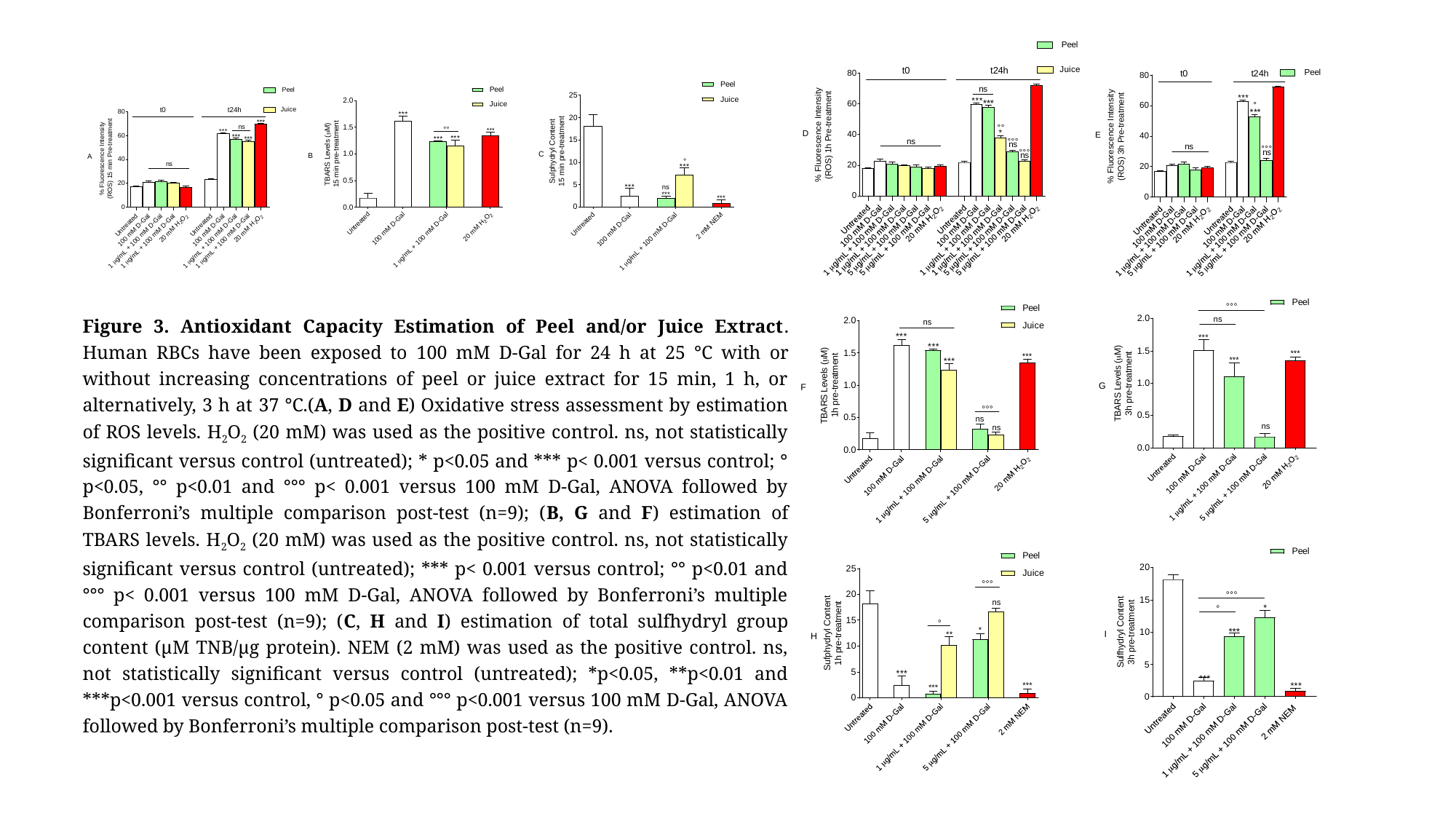

Figure 3. Antioxidant Capacity Estimation of Peel and/or Juice Extract. Human RBCs have been exposed to 100 mM D-Gal for 24 h at 25 °C with or without increasing concentrations of peel or juice extract for 15 min, 1 h, or alternatively, 3 h at 37 °C.(A, D and E) Oxidative stress assessment by estimation of ROS levels. H2O2 (20 mM) was used as the positive control. ns, not statistically significant versus control (untreated); * p<0.05 and *** p< 0.001 versus control; ° p<0.05, °° p<0.01 and °°° p< 0.001 versus 100 mM D-Gal, ANOVA followed by Bonferroni’s multiple comparison post-test (n=9); (B, G and F) estimation of TBARS levels. H2O2 (20 mM) was used as the positive control. ns, not statistically significant versus control (untreated); *** p< 0.001 versus control; °° p<0.01 and °°° p< 0.001 versus 100 mM D-Gal, ANOVA followed by Bonferroni’s multiple comparison post-test (n=9); (C, H and I) estimation of total sulfhydryl group content (µM TNB/µg protein). NEM (2 mM) was used as the positive control. ns, not statistically significant versus control (untreated); *p<0.05, **p<0.01 and ***p<0.001 versus control, ° p<0.05 and °°° p<0.001 versus 100 mM D-Gal, ANOVA followed by Bonferroni’s multiple comparison post-test (n=9).

## Slide 4
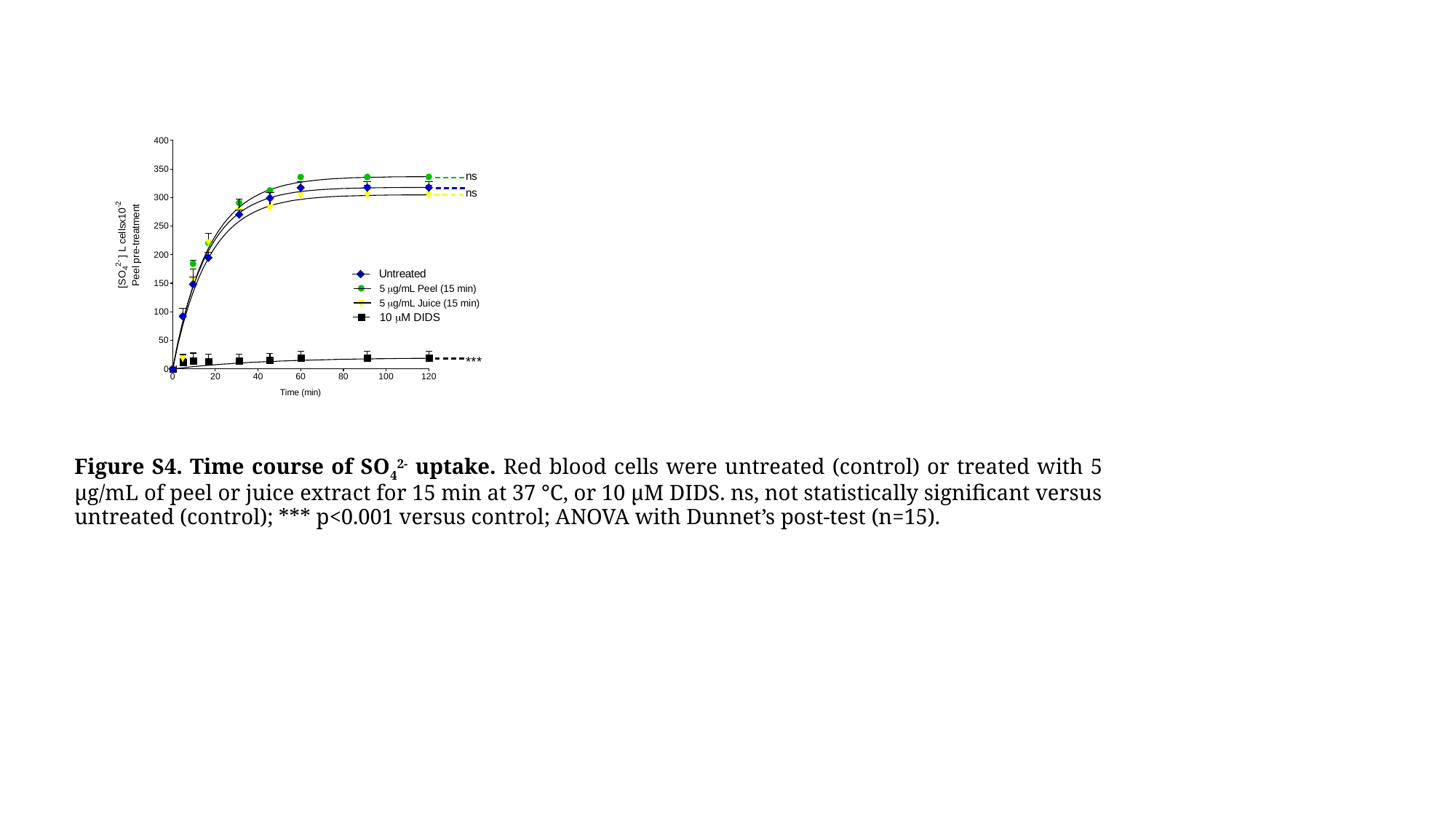

Figure S4. Time course of SO42- uptake. Red blood cells were untreated (control) or treated with 5 µg/mL of peel or juice extract for 15 min at 37 °C, or 10 µM DIDS. ns, not statistically significant versus untreated (control); *** p<0.001 versus control; ANOVA with Dunnet’s post-test (n=15).
